# Noble Metal Nanoparticles Anchored on Transition Metal Phosphides for Effective pH‐Universal Hydrogen Evolution

**DOI:** 10.1002/advs.202504462

**Published:** 2025-04-30

**Authors:** Hang Lei, Yifan Zhou, Zhuowen Huangfu, Liangjun Chen, Jin Cao, Xuelin Yang, Wenjie Mai, Zilong Wang

**Affiliations:** ^1^ Hubei Provincial Collaborative Innovation Center for New Energy Microgrid College of Electrical Engineering & New Energy China Three Gorges University Yichang Hubei 443002 P. R. China; ^2^ Siyuan Laboratory Guangdong Provincial Engineering Technology Research Center of Vacuum Coating Technologies and New Energy Materials College of Physics & Optoelectronic Engineering Jinan University Guangzhou Guangdong 510632 P. R. China; ^3^ Key Laboratory of Advanced Energy Materials Chemistry (Ministry of Education) Nankai University Tianjin 300071 P. R. China

**Keywords:** chemical bonds, electrocatalysts, hydrogen evolution reaction, pH‐universal reactions, Pt nanoparticles

## Abstract

Constructing electrocatalysts with high activity and low precious metal content is essential for achieving efficient hydrogen production in pH‐universal overall water splitting. Herein, five types of noble metal anchored transition metal phosphides are analyzed by theoretical derivation. Based on the calculation results, a suitable hybrid is screened out of Pt nanoparticles anchored on CoP nanowires (Pt─CoP) via robust Pt─P─Co bonds. This strong synergy between Pt and CoP through interfacial Pt─P─Co bonds optimizes the adsorption of key intermediates for hydrogen evolution reaction (HER) in a wide pH range from 0 to 14. Furthermore, strong interaction between Pt and CoP accompanied by a delicate structure reduces interfacial charge transfer resistance, creates abundant active sites, and enhances catalyst durability, while facilitating active site exposure and electron/mass transfer during the HER process. Accordingly, the synthesized Pt─CoP exhibits low overpotentials of 79, 26, and 18 mV at 10 mA cm^−2^ in acidic, neutral, and alkaline media for HER, respectively, superior to commercial Pt/C benchmarks and most reported electrocatalysts. This work paves a new clue to exploit electrocatalysts with low‐Pt‐loading for pH‐universal HER.

## Graphical abstract

Seven types of noble metal‐transition metal phosphide hybrids are analyzed through theoretical calculations. Based on these theoretical results, a promising Pt‐CoP hybrid is prepared, consisting of strongly coupled Pt nanoparticles and CoP nanowires connected by robust Pt─P─Co bonds. The synthesized Pt─CoP catalyst demonstrates exceptional mass activity and long‐term stability in a pH‐universal hydrogen evolution reaction.

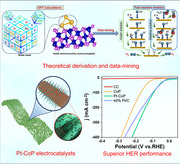

## 1 Introduction

Green hydrogen from water electrocatalysis, powered by intermittent renewable energy, has given significant opportunities to achieve the strategic goals of renewable energy storage and carbon neutrality.^[^ ^1^, ^2^ ^]^ The hydrogen evolution reaction (HER) can be conducted in acidic, neutral, and alkaline conditions, which plays a crucial part in electrochemical water splitting and dominates the hydrogen production efficiency.^[^ ^3^, ^4^ ^]^ However, although tremendous effectors have been made, the sluggish kinetics and different reaction mechanisms of HER still significantly increase energy consumption, especially at large current densities in neutral/alkaline conditions.^[^ ^5^, ^6^, ^7^ ^]^ Pt/C is considered as the benchmark electrocatalyst for HER, while the low reserve and fancy price limit its large‐scale application. It is therefore of great technological and industrial significance to exploit the low noble metal loading electrocatalysts with high activity and stability which can operate well in pH‐universal HER.^[^ ^8^, ^9^ ^]^

Recently, hydrogen evolution induced by the interactions between metal clusters/nanoparticles and transition metal oxide/phosphide/MOF supports have been regarded as promising solutions to facilitate the HER kinetics and reduce noble metal usage in electrocatalysts.^[^ ^10^, ^11^, ^12^, ^13^ ^]^ However, how to lower the thermodynamic obstacles and optimize the adsorption of intermediates at the metal‐support interface is one of the most critical challenges to developing pH‐universal HER catalysts.^[^ ^14^, ^15^ ^]^ Previous experimental results and theoretical calculations indicate that noble metals anchored on transition metal phosphide (TMP) can construct interfacial noble metal‐heteroatom‐transition metal bonds, which optimize the hydrogen binding energy and accelerate the kinetics in HER, resulting in satisfactory performance and low noble metal usage.^[^ ^16^, ^17^ ^]^ For instance, Wang et al. demonstrated an anion‐deficient strategy that enables the precise immobilization of Ru single atoms within the anion vacancies of CoP. These Ru single atoms preferentially anchor at the anion vacancy sites, thereby constructing a highly efficient catalytic surface with neighboring Co─P and Co─Ru coordination environments, which significantly enhances the performance of HER catalysis.^[^ ^18^ ^]^ Liu et al. reported the development of heterogeneous Ru─CoP urchin arrays on carbon cloth (Ru─CoP/CC), guided by density functional theory (DFT) calculations. Their study revealed that a strong built‐in electric field within the Ru─CoP/CC structure significantly reduced the barrier for hydrogen spillover from the interfacial Ru sites to the Co sites, which exhibited near‐zero hydrogen adsorption energy.^[^ ^19^ ^]^ It is believed that the incorporation of noble metals can significantly promote the electrocatalytic properties of TMP‐based materials. However, developing a comprehensive theory to design effective electrocatalysts of noble metal on TMPs supports for pH‐universal HER remains a significant challenge.

Herein, guided by theoretical derivation, first‐principles simulations, and data mining, we establish a general theory to explore the strong coupled noble metal and TMPs hybrid electrocatalysts (N‐TMPs, N stands for Pt, Au, Ir, Pd, In) for pH‐universal hydrogen production. Based on the DFT calculation results of various types of N‐TMPs hybrid, we confirm that a combination of Pt nanoparticles and CoP species through Pt─P─Co bonds can effectively optimize water adsorption/dissociation and the hydrogen adsorption free energy, which is beneficial to lower the reaction energy barrier and improve pH‐universal HER kinetics. Among all the N‐TMPs, the Pt─CoP exhibits the favorable d band center (𝜺_d_) value (−2.45 eV) and the most moderate intermediate H adsorption free energy (ΔG_H*_) value (−0.04 eV). Under the guidance of theoretical calculation results, a series of N‐TMPs were fabricated successfully by anchoring noble metal on TMPs. Specially, the hybrid of Pt nanoparticles anchored on CoP nanowires by Pt─P─Co bonds can accelerate the interfacial charge transfer and provide a more negative integrated crystal orbital hamilton population (ICOHP) value with a low Pt loading. Benefiting from the moderate ΔG_H*_ value, the lower energy barrier for H_2_O dissociation, and stable Pt─P─Co coordination, the synthesized Pt─CoP exhibits excellent catalytic activity and long‐term stability at pH‐universal HER. Our work offers a new generalized strategy for designing noble metal‐transition metal phosphide hybrids for pH‐universal electrocatalytic hydrogen evolution.

## 2 Results and Discussion

**Figure 1:**
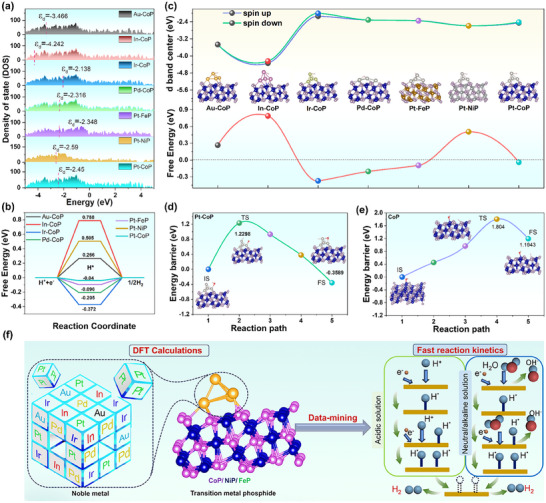
a) TDOS of various noble metal‐TMPs hybrid, the 𝜺_d_ value is assigned to the d‐band center of the metal cluster in the hybrids. b) The intermediate H adsorption free energy diagram. c) The relationship between the 𝜺_d_ value and ΔG_H*_. The kinetic energy barrier for H_2_O dissociation on d) Pt─CoP surface and e) CoP surface. f) Schematic illustration of DFT calculations used to guide the screening of N‐TMPs catalysts.

In a pH‐universal HER system, the reaction behavior and mechanism differ due to the diverse proton sources in acidic, alkaline, and neutral electrolytes during the catalytic process.^[^ ^20^ ^]^ The water adsorption/dissociation and the intermediate H adsorption free energy (ΔG_H*_) are widely applied as important activity descriptors to forecast the potentiality of electrocatalyst for pH‐universal HER.^[^ ^21^, ^22^ ^]^ Herein, the DFT calculations were employed to probe the thermodynamic and kinetic behavior throughout the pH‐universal HER process in order to guide the design of N‐TMPs. Generally, the type of metal nanoparticles substantially impacts their adsorption characteristics, which can subsequently modify the intrinsic HER activity.^[^ ^23^ ^]^ Hence, five types of common noble metals (Pt, Pd, Au, Ir, In) and the optimal crystal plane (111) of common TMPs (CoP, NiP, and FeP) were chosen to examine the influence of noble metal species on HER activity. The calculation models for noble metals and TMPs hybrids are depicted in Figure S1 (Supporting Information) and the noble metal atoms are anchored to the phosphide surface by bonding with phosphorus. Firstly, the density of states (DOS) of various noble metal‐TMPs hybrid was calculated (Figures S2 and S3, Supporting Information). The 𝜺_d_ value originates from the d‐band center of the metal cluster in the structure, which is attributed to the fact that the H intermediate adsorption sites are mainly located on the metal cluster. The upshift of the d‐band center will make the bonding orbitals more likely to be occupied by molecules and in turn, enhance the hydrogen adsorption energy.^[^ ^24^ ^]^ Similarly, the more negative the 𝜺_d_ value, the higher the probability that the bonding orbitals are filled with electrons, and thus the lower the probability that molecules will occupy these orbitals, resulting in a weaker adsorption energy. As shown in **Figure** **1**a, analysis of DOS reveals that the Pt cluster anchored on CoP has the most favorable 𝜺_d_ of −2.45 eV compared to Au─CoP, In─CoP, Ir─CoP, Pd─CoP, Pt─FeP, Pt─NiP, indicating Pt─CoP has the most suitable H intermediate adsorption strength. Furthermore, the ΔG_H*_ in the hybrid system was also explored (Figure 1b). Among all seven types of noble metal‐TMPs hybrid, the Pt─CoP presents a ΔG_H*_ value closest to 0. In accordance with the Sabatier principle, an optimal ΔG_H*_ value close to zero is conducive to hydrogen adsorption and desorption in the HER process.^[^ ^25^ ^]^ The ΔG_H*_ value (−0.04 eV) of Pt─CoP indicates more facile hydrogen adsorption and desorption, thus resulting in higher catalytic activity for HER.

Based on DFT calculations, the relationship between 𝜺_d_ and ΔG_H*_ is established for these seven types of noble metal‐TMPs hybrid in Figure 1c. Notably, the Pt─CoP exhibits the favorable 𝜺_d_ value (−2.45 eV) and the most moderate ΔG_H*_ value (−0.04 eV) among the seven types of N‐TMP hybrid. This can be explained through the electron‐interaction model, where bonding and antibonding states are created between the adsorbate's valence p‐level and the metal's d‐band.^[^ ^26^ ^]^ The antibonding states lead to Pauli repulsion, which in turn reduces bond strength.^[^ ^27^ ^]^ Furthermore, an upshift of the d‐band center results in more bonding states below the Fermi level, thereby enhancing the bond strength. Briefly, the combination of Pt and CoP exhibits an appropriate d band center and the most moderate ΔG_H*_ value that is beneficial for achieving highly active HER. Based on the above‐mentioned analysis, we chose Pt─CoP as the focal subject for a more in‐depth examination of the kinetic and thermodynamic behaviors associated with water molecule dissociation on its surface. Figure 1d,e indicates the reaction paths for the first step in the dissociation of the water molecule on Pt─CoP and CoP, respectively. The initial state (IS) with the related energy set as 0, and transition state denoted as TS and the final state denoted as FS. The lower energy barrier for H_2_O dissociation is 1.229 eV in Pt─CoP (Figure 1d), whereas the energy barrier for H_2_O dissociation on the active sites of CoP is 1.804 eV (Figure 1e). The lower kinetic energy barrier of Pt─CoP means a proton can be preferentially generated on Pt─CoP compared to the CoP surfaces in dynamics.^[^ ^28^ ^]^ The energy from IS to FS of Pt─CoP (−0.358 eV) is also much smaller than that of CoP (1.194 eV), indicating that the dissociation of the water molecule on the Pt─CoP surface is a preferred thermodynamic behavior.^[^ ^29^ ^]^ The low kinetic energy barrier and suitable thermodynamic driving force of Pt─CoP emphasize the important role of synergy between Pt and CoP in optimizing the interaction between the catalyst and the water molecules/intermediates. These results suggest that Pt‐anchored CoP enables optimum water adsorption/dissociation and hydrogen adsorption‐free energy, which synergistically leads to reduce the reaction energy barrier and elevated pH‐universal HER kinetics (Figure 1f).^[^ ^30^, ^31^, ^32^ ^]^

**Figure 2:**
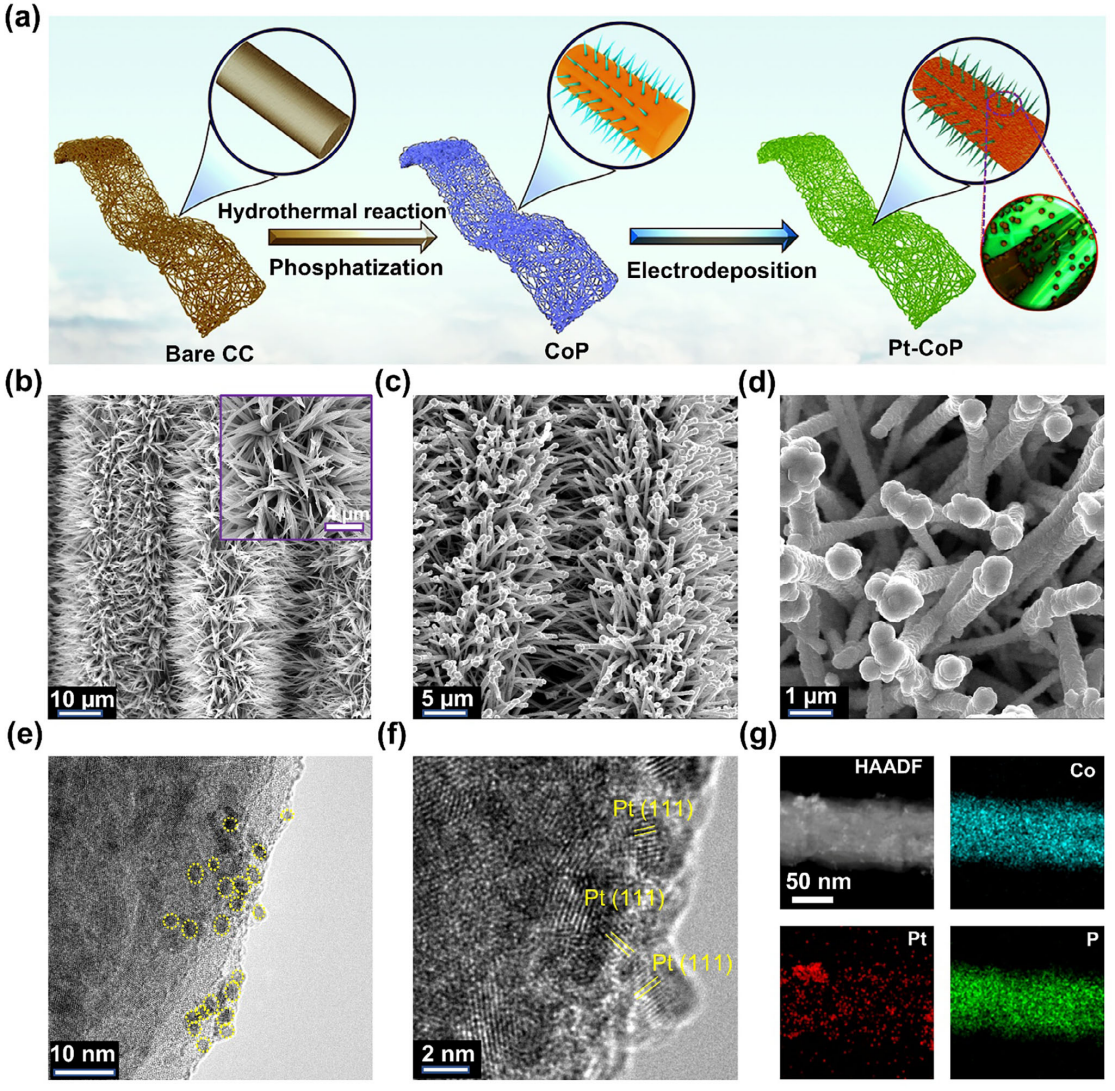
a) The schematic illustration of the synthetic procedures for Pt─CoP. b) The SEM image of CoP nanowires. c,d) The SEM images of Pt─CoP nanorods at different resolutions. e,f) High‐resolution TEM image of Pt─CoP. g) HAADF‐STEM images and corresponding elemental mapping images of Pt─CoP.

The results of theoretical calculations strongly demonstrate that the combination of Pt and CoP can achieve efficient pH‐universal HER. As a verification, we anchored Pt nanoparticles on the surface of CoP nanowires (denoted as Pt─CoP) through a simple method. The preparation process of Pt─CoP is shown in **Figure** **2**a, and specific preparation details can be found in the experimental section of the supporting information. The structural transformations from the initial carbon cloth (CC) to the final Pt─CoP product were characterized using scanning electron microscopy (SEM) and transmission electron microscopy (TEM). The surface of the original CC appears bare because there is no load (Figure S4, Supporting Information). After hydrothermal treatment, Co(OH)F grows in situ on the surface of the CC, forming an ordered nanowire array (Figure S5, Supporting Information). The CoP still maintains a highly ordered nanowire array structure after phosphorization (Figure 2b); however, after the Pt nanoparticles were electrodeposited on the surface of CoP, the morphology of the catalyst transformed from nanowires to flammulina enoki‐like nanorods (Figure 2c,d). TEM image shows that Pt nanoparticles are dispersed randomly on the surface of CoP (Figure 2e). High‐resolution TEM images show that Pt nanoparticles tend to expose the Pt (111) crystal plane (Figure 2f). The high‐angle annular dark‐field scanning transmission electron microscopy (HAADF‐STEM) image also confirmed that ultrafine Pt nanoparticles were dispersed disorderly on the surface of CoP (Figure 2g). The corresponding energy dispersive X‐ray (EDX) spectra show the distribution of Co, P, and Pt elements on the Pt─CoP nanorod. The morphological analyses confirmed that Pt nanoparticles were successfully anchored on the CoP surface, forming flammulina enoki‐like nanorod arrays.

**Figure 3:**
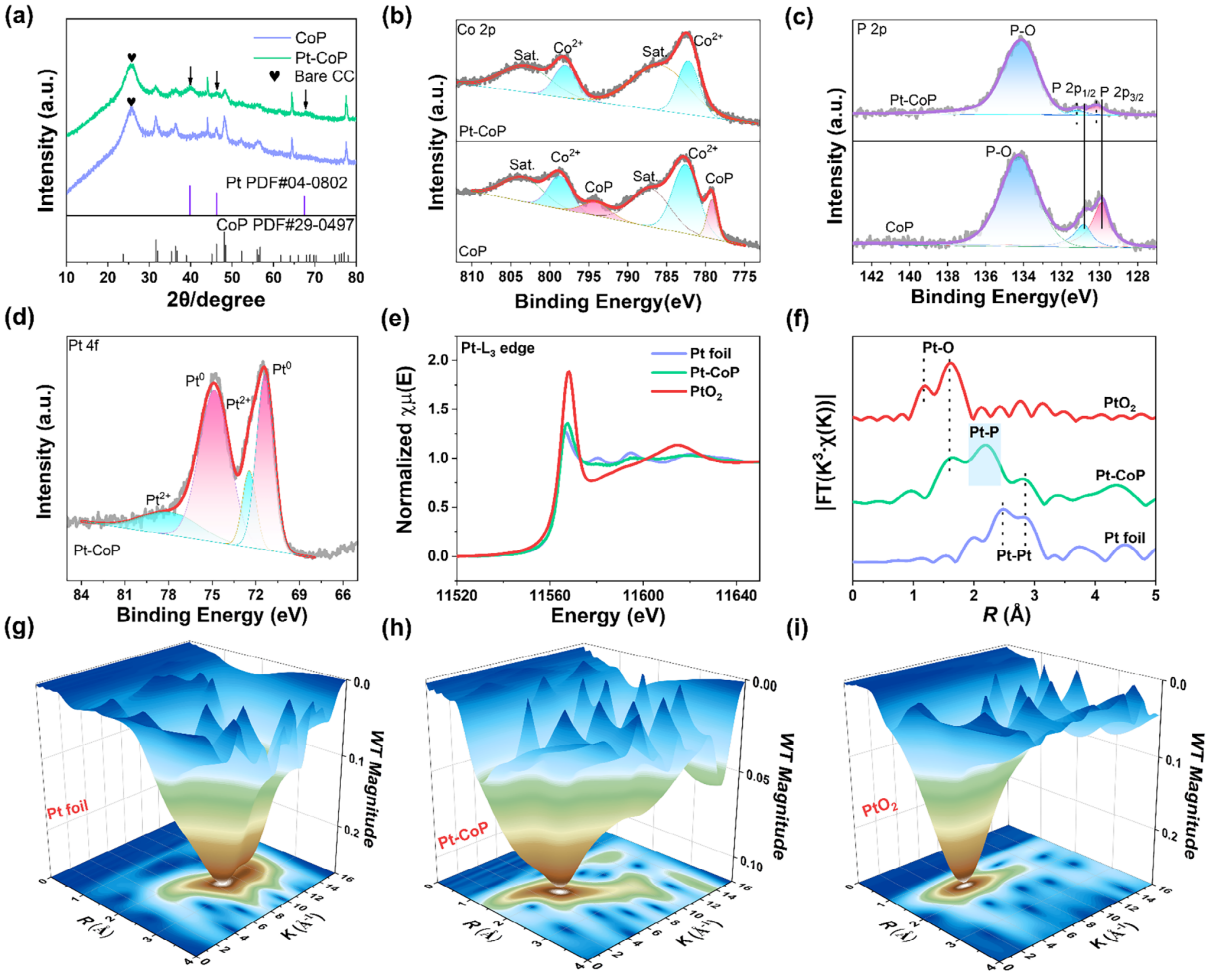
a) The XRD pattern of Pt─CoP and CoP. High‐resolution XPS spectra of b) Co 2p, c) P 2p, and d) Pt 4f. e) XANES spectra for Pt─CoP, Pt foil, and PtO_2_. f) Fourier transformed EXAFS spectra. g–i) WT images.

Subsequently, X‐ray powder diffraction (XRD) patterns and X‐ray photoelectron spectroscopy (XPS) were employed to investigate the composition and valence states of the as‐prepared catalysts and the interactions between Pt and CoP. In the XRD pattern of Pt─CoP (**Figure** **3**a), in addition to the characteristic peaks of CoP (PDF#29‐0497) and CC, the characteristic peaks at 39.8°, 46.5°, and 67.9° correspond to (111), (200), and (220) planes of Pt (PDF#04‐0802). There is a phenomenon that the characteristic peaks of Pt and CoP overlap at 46.5°. Due to the surface coverage effect of Pt nanoparticles, the intensity of the peak at 46.5° for Pt─CoP is weaker than that for pure CoP.^[^ ^33^ ^]^ In Co 2p XPS spectra (Figure 3b), the characteristic peaks at 779.1 and 794.1 eV belong to Co─P, while peaks at 781.47 and 797.67 eV are attributed to Co^2+^.^[^ ^34^, ^35^ ^]^ In P 2p XPS spectra (Figure 3c; Figure S6, Supporting Information), there are three characteristic peaks located at 134.2, 130.8, and 129.9 eV, which are attributed to P─O, P 2p_1/2_ and P 2p_3/2_ of Co─P bond, respectively.^[^ ^36^ ^]^ After anchoring Pt nanoparticles, the intensity of the Co─P peaks decreases, while the integral area ratio of the P─O peak increases. This phenomenon is likely attributed to the coating effect of the Pt nanoparticles. The slight shift of the P 2p peak of Pt─CoP confirmed the strong electron interactions between Pt nanoparticles and CoP nanowires.^[^ ^37^ ^]^ For Pt─CoP, the peaks of Pt 4f (Figure 3d) located at 74.8, 71.4, 78.4, and 72.5 eV are ascribed to Pt^0^ 4f_5/2_, Pt^0^ 4f_7/2_, Pt^2+^ (PtO) 4f_5/2_, and Pt^2+^ (PtO) 4f_7/2_, respectively.^[^ ^38^ ^]^

To further identify the interaction between Pt and CoP, the X‐ray absorption near edge structure (XANES), extended X‐ray absorption fine structure (EXAFS), and wavelet transform (WT) analysis were investigated to reveal how the Pt nanoparticles modification regulates the local coordination environment and electronic structure of Pt─CoP.^[^ ^39^ ^]^ In the XANES at the Pt L_3_‐edge of Pt─CoP and references (Pt foil and PtO_2_), the white‐line peak of Pt─CoP is between that of Pt foil and PtO_2_, indicating Pt atoms carry positive charges and possess a cationic environment around Pt atoms (Figure 3e).^[^ ^10^, ^40^ ^]^ In EXAFS spectra (Figure 3f), compared with Pt foil and PtO_2_, Pt─CoP contains an obvious characteristic peak (2.17 Å) in addition to the Pt─O and Pt─Pt peaks. Given that oxygen, O is more electronegative than phosphorus P, and P in this system coordinates with both platinum Pt and cobalt Co, the prominent peak at 2.17 Å can be ascribed to the charge redistribution induced by the Pt─P─Co coordination. This observation indicates the presence of strong electronic interactions and couplings between Pt nanoparticles and CoP nanowires.^[^ ^41^, ^42^ ^]^ The strong coupling effect between Pt nanoparticles and CoP nanowires may facilitate charge transport at the interface, optimize the adsorption and desorption of intermediates, and improve the long‐term stability of electrocatalysts at high current densities. Furthermore, WT analysis (Figure 3g–i) provides the overlapping contributions in the radial distance at R‐ and K‐space, which more strongly confirms that Pt nanoparticles are anchored on CoP nanowires through the Pt─P─Co bridge bond.^[^ ^43^ ^]^ Hence, based on the results of the X‐ray absorption spectrum, it could be concluded that the combination of Pt nanoparticles and CoP nanowires by Pt─P─Co bonds can effectively regulate the local electronic structure and coordination environment of Pt atoms.

**Figure 4:**
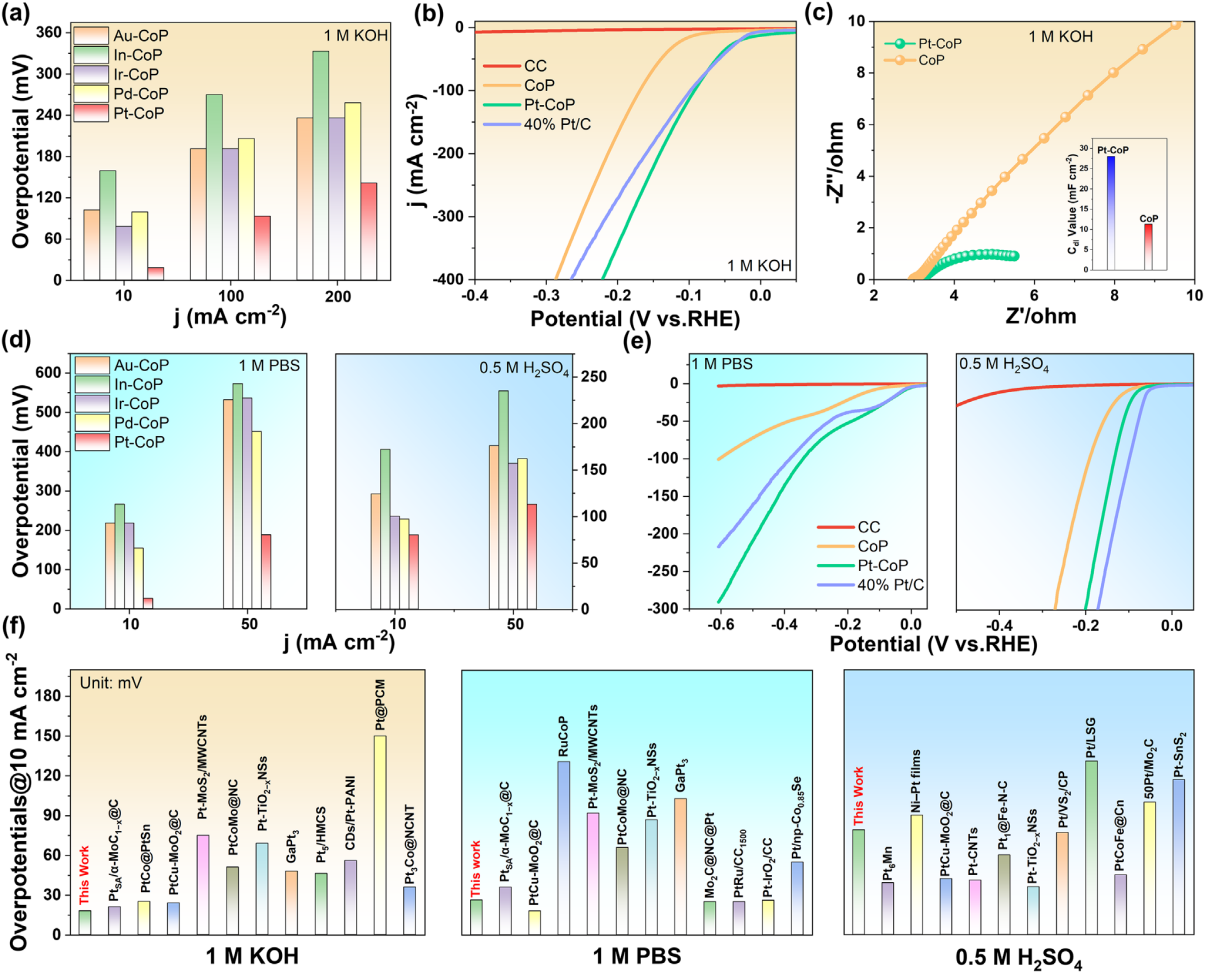
a) Comparison of the catalytic activities of various noble metals and TMPs hybrids for HER in 1 M KOH solution. b) Linear sweep voltammetry (LSV) curves of a variety of catalysts for the HER. c) Nyquist plots and electrochemical surface areas (inset) of Pt─CoP and CoP. d) Comparison of the catalytic activities of various noble metals and TMPs hybrid for HER in 1 M PBS and 0.5 M H_2_SO_4_ solution. e) HER polarized curves of various catalysts in 1.0 M PBS and 0.5 M H_2_SO_4_ solutions. f) Comparisons of HER overpotentials at 10 mA cm^−2^ with other reported Pt‐based catalysts.

The electrocatalytic performances of various noble metals and TMPs hybrid toward HER were first investigated in a 1.0 m KOH solution. As shown in **Figure** **4**a, the overpotential of Pt─CoP at various current densities (10–200 mA cm^−2^) is much lower than that of other N‐TMPs hybrid catalysts, indicating that Pt─CoP has higher catalytic activity and faster reaction kinetics. Furthermore, the Pt─CoP also exhibits the best catalytic activity compared to bare CC, CoP, and commercial Pt/C, especially at high current density (Figure 4b). The Pt─CoP shows a near‐zero onset potential, and the overpotential at a current density of 10 mA cm^−2^ is only 18 mV, indicating that Pt─CoP undergoes rapid dissociation of the water molecule, which is consistent with the DFT calculation results. Electrochemical impedance spectroscopy (EIS) reveals that Pt─CoP exhibits a smaller charge‐transfer resistance (R_ct_) compared to CoP (Figure 4c), indicating enhanced electron transfer efficiency and improved reaction kinetics during the HER process. Additionally, the electrochemically active surface area (ECSA) of Pt─CoP is significantly larger than that of CoP after modification with Pt nanoparticles (inset in Figure 4c). This larger ECSA and smaller R_ct_ of Pt─CoP facilitate electron transfer and optimize exposure of active sites, thereby accelerating the HER kinetics. The catalytic properties of various N‐TMP catalysts were also investigated in both neutral (1 m phosphate‐buffered saline electrolyte, PBS) and acidic (0.5 M H₂SO₄) media. Among them, Pt─CoP demonstrated the lowest overpotentials at current densities of 10 and 50 mA cm⁻^2^, highlighting its superior HER activity across a broad pH range. Specifically, in neutral electrolytes, Pt─CoP outperformed 40% Pt/C, exhibiting an overpotential of only 26.3 mV at 10 mA cm⁻^2^. In acidic electrolytes, Pt─CoP still exhibited excellent catalytic activity, with an overpotential of just 79 mV at 10 mA cm⁻^2^ which was slightly inferior to that of 40% Pt/C. Based on the inductively coupled plasma‐mass spectrometry (ICP‐MS) results, the atomic ratio of Pt in Pt─CoP is 3.59% (Figure S7, Supporting Information), and the mass activities of Pt─CoP and 40% Pt/C were further explored. Notably, the mass activity of Pt─CoP in alkaline, neutral, and acidic electrolytes significantly outperforms that of 40% Pt/C, demonstrating that Pt nanoparticles anchored on CoP can effectively reduce the Pt loading while enhancing its intrinsic catalytic activity (Figures S8–S10, Supporting Information). Strikingly, the overpotentials of Pt─CoP at a current density of 10 mA cm^−2^ are far ahead of that of previously reported Pt‐based electrocatalysts, and it also maintains an advanced level under full pH range (Figure 4f; Tables S1–S3, Supporting Information).

**Figure 5:**
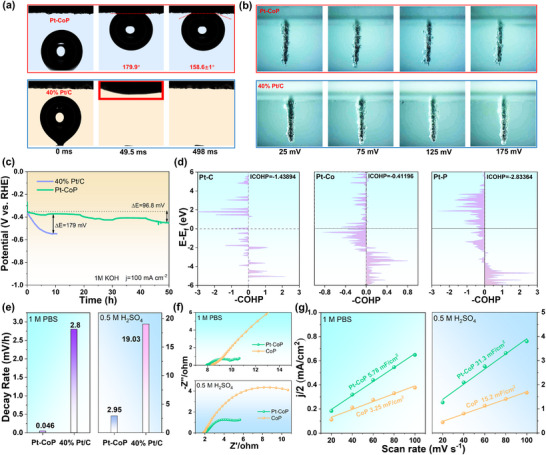
a) Adhesion behaviors of hydrogen bubbles and the corresponding digital images of hydrogen bubbles on Pt─CoP and 40% Pt/C. b) Digital photos demonstrate the hydrogen release behaviors of Pt─CoP and 40% Pt/C. c) Chronopotentiometry curves at a constant current density of 100 mA cm^−2^ for Pt─CoP and 40% Pt/C in 1.0 m KOH solution. d) The crystal orbital hamilton population analysis of Pt─C bond, Pt─Co bond, and Pt─P bond. e) The HER potential decay rate in 1 m PBS and 0.5 m H_2_SO_4_ solutions. f) Nyquist plots and g) electrochemical surface areas of Pt─CoP and CoP in 1.0 m PBS and 0.5 m H_2_SO_4_ solutions.

Given the critical role of hydrogen bubble evolution in influencing the reactivity of active sites and intermediates/reactants, we conducted a detailed analysis of the hydrogen bubble adhesion properties on Pt─CoP and 40% Pt/C electrodes by measuring their contact angles. As displayed in **Figure** **5**a, Pt─CoP exhibits significantly larger contact angles compared to 40% Pt/C, indicating reduced adhesion of hydrogen bubbles to the electrode surface. In contrast, the 40% Pt/C electrode, readily adsorbs hydrogen bubbles. Further investigation of hydrogen bubble evolution behavior under varying overpotentials (25–175 mV) revealed distinct differences between the two electrodes. As shown in Figure 5b, the 40% Pt/C electrode surface tends to accumulate a gas film, impeding bubble desorption regardless of the overpotential magnitude. Conversely, hydrogen bubbles on the Pt─CoP electrode detach more readily, facilitating efficient bubble release. The systematic comparative analysis of hydrogen bubble adhesion and release dynamics on Pt─CoP nanoarray electrodes versus 40% Pt/C flat electrodes reveals distinct differences. The nanoarray architecture of Pt─CoP demonstrates significantly reduced bubble adhesion forces and accelerated desorption kinetics, directly attributable to its hierarchical surface morphology. These structural advantages minimize gas bubble residence time while simultaneously increasing active site accessibility. The synergistic effects of enhanced mass transfer efficiency reduced ohmic losses, and optimized charge transfer kinetics collectively bolster the superior electrocatalytic performance of Pt─CoP.^[^ ^44^, ^45^, ^46^ ^]^ Moreover, Pt─CoP demonstrates remarkable stability in alkaline solutions. When subjected to a constant current density of 100 mA cm⁻^2^ for 50 h (Figure 5c), the operating potential of Pt─CoP increased by only 96.8 mV, corresponding to a potential decay rate of 1.94 mV h^−1^. In contrast, the operating potential of Pt/C increased by 179 mV after just 10 h of testing, with a potential decay rate of 17.9 mV h^−1^. To elucidate the origin of the superior stability of Pt─CoP over 40% Pt/C, the crystal orbital hamilton population (COHP) technique calculations were performed to explore the Pt─C, Pt─Co, and Pt─P bond formation between the Pt and the carrier (C and CoP). The COHP diagram partitions the density of states into bonding, nonbonding, and antibonding contributions. Within the energy domain situated below the Fermi level (E_f_), an elevated contribution from the bonding state is directly correlated with an enhancement in the stability of the chemical bond.^[^ ^12^, ^47^ ^]^ Conventionally, more negative ICOHP values are indicative of stronger bonds, thus serving as a valuable metric for assessing the stability and character of individual chemical bonds.^[^ ^48^, ^49^ ^]^ As shown in Figure 5d, despite the relatively low ICOHP value (−0.411) observed for Pt─Co bond, the ICOHP value of Pt─P (−2.833) significantly surpasses that of Pt─C (−1.438). This suggests that the bond formed between Pt and P atoms within CoP is more efficient than the bond between Pt and C atoms in Pt/C, indicating greater stability in the Pt─P bond. These calculation results confirm from the perspective of bonding that the combination of Pt and CoP through Pt─P─Co bonds can provide a more stable chemical state during HER operation, thus improving the stability of the catalyst, especially at high current densities. To confirm the excellent stability of Pt─CoP, we performed TEM and XPS analyses after subjecting the Pt─CoP sample to continuous testing at a current density of 50 mA cm^−2^ for 50 h in an alkaline solution. As illustrated in Figure S11 (Supporting Information), the Pt─CoP maintained its overall nanorod structure after the stability test, with Pt nanoparticles remaining individually dispersed on the CoP nanorods without any signs of agglomeration. Moreover, XPS analysis (Figure S12, Supporting Information) revealed that the valence state and surface composition of Pt─CoP remained largely unchanged, with no emergence of new species. These results collectively demonstrate that Pt─CoP exhibits exceptional stability in both nanostructure and phase composition. Significantly, Pt─CoP also exhibits exceptional long‐term stability in both acidic and alkaline environments. As illustrated in Figure 5e and Figure S13 (Supporting Information), in 1 M PBS solution, Pt─CoP demonstrates a low potential decay rate of only 0.046 mV h^−1^, compared to 2.8 mV h^−1^ for 40% Pt/C. Similarly, in 1 M H₂SO₄ solution, Pt─CoP maintains a notably low potential decay rate of 2.95 mV h^−1^. Figure 5f,g further confirms that Pt─CoP has a lower electron transfer resistance and a larger electrochemically active surface area than CoP, regardless of whether the conditions are acidic or alkaline.

Based on the results of theoretical calculations, systematic characterization, and electrochemical measurements, it can be concluded that the combination of CoP nanowires with Pt nanoparticles represents an effective strategy for synthesizing electrocatalysts capable of efficient HER performance across all‐pH media. The excellent electrocatalytic HER performance of Pt─CoP in all‐pH range can be attributed to the following points: i) Modification of Pt nanoparticles on CoP greatly optimizes water adsorption/dissociation and the hydrogen adsorption free energy, thus lowering the reaction energy barrier and improving pH‐universal HER kinetics;^[^ ^50^, ^51^ ^]^ ii) the interaction between Pt nanoparticles and CoP not only reduces the interfacial charge transfer resistance but also enhances the stability of Pt─CoP in pH‐universal HER through the strong Pt─P bond;^[^ ^52^ ^]^ iii) The 3D self‐supporting scaffold constructed by ultra‐fine Pt nanoparticles loaded on ordered CoP nanowire arrays not only facilitates the exposure of active sites and provides abundant reaction sites, but also accelerates the diffusion of electrolytes/protons/intermediates and release of hydrogen gas bubbles.^[^ ^53^ ^]^

## 3 Conclusion

In this work, we analyzed the H adsorption free energy and d‐band center of various noble metal nanoparticles coupled TMPs by DFT calculation. Results of theoretical simulations indicate that Pt nanoparticles can optimize the free energy and kinetic process for pH‐universal hydrogen evolution. Guided by theoretical analysis, we synthesized a strongly coupled Pt─CoP hybrid, obtaining excellent catalytic properties for hydrogen evolution over a wide range of pH values. The Pt─P─Co bonds efficiently diminish interfacial impedance and significantly amplify the coupling strength between these two components. The ordered flammulina enoki‐like nanorods arrays enhance the diffusion of electrolytes/proton/intermediates and release of hydrogen gas bubbles. Accordingly, the synthetic Pt─CoP catalyst delivers remarkable mass activity and long‐term durability at a pH‐universal HER system, which is superior to the state‐of‐the‐art 40% Pt/C. This work highlights the construction of strong chemical bonds between heterogeneous phases to enhance the activity and stability of HER electrocatalysts.

## Conflict of Interest

The authors declare no conflict of interest.

## Author Contributions

H.L. conceived the idea and carried out the experiment. Y.F.Z., Z.W.H., L.J.C., and J.C. evaluated the electrochemical performances and analyzed the structural characterization. Z.L.W., W.J.M., and X.L.Y. supervised the whole work. All authors co‐discussed the results and wrote the manuscript.

## Supporting information



Supporting Information

## Data Availability

The data that support the findings of this study are available from the corresponding author upon reasonable request.
