# Supplementary material for: Noble Metal Nanoparticles Anchored on Transition Metal Phosphides for Effective pH‐Universal Hydrogen Evolution
Source: Adv Sci (Weinh). 2025 Apr 30;12(27):2504462. doi: 10.1002/advs.202504462 (PMC12279237; doi:10.1002/advs.202504462)
Supplement: Supplementary file 1 — Supporting Information [file ADVS-12-2504462-s001.docx]

**Supporting Information**

**Noble metal nanoparticles anchored on transition metal phosphides for effective pH-universal hydrogen evolution**

*Hang Lei, Yifan Zhou, Zhuowen Huangfu, Liangjun Chen, Jin Cao, Xuelin Yang, Wenjie Mai,* *Zilong Wang**

[a] H. Lei, Y. F. Zhou, J. Cao, Prof. X. L. Yang

Hubei Provincial Collaborative Innovation Center for New Energy Microgrid, College of Electrical Engineering & New Energy

China Three Gorges University

Yichang 443002, Hubei, P.R. China

[b] Z. W. Huangfu, L. J. Chen, Prof. Z. L. Wang, Prof. W. J. Mai

Siyuan Laboratory, Guangdong Provincial Engineering Technology Research Center of Vacuum Coating Technologies and New Energy Materials, College of Physics & Optoelectronic Engineering

Jinan University

Guangzhou, Guangdong, P. R. China

E-mail: zilong@email.jnu.edu.cn

[c] H. Lei,

Key Laboratory of Advanced Energy Materials Chemistry (Ministry of Education), Nankai University

Tianjin 300071, P. R. China

**1. Experimental Section**

**1.1 Pre-treatment of carbon cloth**

Firstly, the carbon cloths (CC) were soaked in 1M hydrochloric acid under 120 ℃ for 4 h to enhance its hydrophilicity, and then they were washed with ethanol, acetone, and deionized water under vigorous stirring for 10 mins in sequence. After that, the carbon cloth was dried at 60 ℃ for later use.

**1.2 Preparation of Co(OH)F**

Firstly, 1 mmol Co(NO_3_)_2_·6H_2_O, 3 mmol NH_4_F, and 6 mmol urea were dissolved in 20 ml deionized water and stirred for 30 mins. The CC was cut into the size of 2 cm × 3 cm, and the top of the CC small pieces were fixed on a polytetrafluoroethylene board. Then, the as-prepared solution and CC pieces were transferred to a 50 ml Teflon high-temperature reactor. The CC pieces were vertically immersed in the solution and kept at 120 ℃ for 6 h in the oven. When the hydrothermal was cooled to room temperature, the CC pieces were washed with deionized water several times, and let them dry at 60 ℃ overnight.

**1.3 Preparation of CoP**

Firstly, the as-obtained Co(OH)F and excessive NaH_2_PO_2_·H_2_O (1 g) were put into two detached corundum boats. After that, the samples were calcined at 300 °C for 2 h at a heating rate of 2 °C·min^–1^ under an argon atmosphere. Finally, the CoP was obtained after the tube furnace was naturally cooled to room temperature.

**1.4 Preparation of Pt-CoP**

Pt-CoP was prepared by electrochemical deposition. Firstly, 1 g of H_2_PtCl_6_·6H_2_O and 0.19 g Na_3_C_6_H_5_O_7_·H_2_O were added into 250 ml deionized water and stirred to form a homogeneous solution. The pH value was adjusted to about 7.0 by adding Na_2_CO_3_, and the obtained solution was used as electrodeposited Pt sources. A three-electrode system consists of CoP, carbon rod, and Ag/AgCl as the working electrode, counter electrode, and reference electrode, respectively. Next, the cyclic voltammetry (CV) was performed at the applied voltage range was -0.8~0.4 V vs. Ag/AgCl, the sweep rate was 50 mV/s, and this electrodeposition was carried out at ten CV cycles at the temperature of 60 ℃. The prepared samples were washed with deionized water several times and dried at 60 ℃ overnight. The Pt loadings were adjusted by varying the CV cycles in the synthesis process. For simplicity, Pt-CoP with 10 CV was used to represent Pt-CoP in this work unless otherwise mentioned. The synthesis routes of other types of noble metal-CoP are similar to those of Pt-CoP, except that the sources of the noble metals are different.

**1.5 Preparation of commercial 40% Pt/C electrode**

5 mg commercial 40% Pt/C and 5 mg carbon black were dispersed into the mixed solution of 980 μL ethanol and 20 μL Nafion solution. A uniform ink was formed after ultrasound for 30 minutes. 100 μL ink was dropped onto 1 cm × 1cm CC and dried at 60 ℃.

**1.6 Characterization**

The microstructure of the prepared catalyst was observed by field-emission scanning electron microscopy (FE-SEM, ZEISS ULTRA 55) and transmission electron microscopy (TEM, FEI Talos F200x). The chemical composition and valence state of the catalyst were analyzed by X-ray diffractometer (XRD, Rigaku, MiniFlex600) and X-ray photoelectron spectroscopies (XPS, Thermo Fisher Scientific, K-Alpha). The Pt L-edge X-ray absorption fine structure (XAFS) spectra were obtained at the Synchrotron Radiation Laboratory in Beijing, China, and the measurement mode used was fluorescence mode. The elemental composition of Pt was measured by Inductively Coupled Plasma Mass Spectrometry (ICPMS, Agilent 7700).

**1.7 Electrochemical measurements**

All electrochemical measurements were performed on the Chenhua CHI 660E electrochemical workstation with a three-electrode system at room temperature. 1 M KOH (pH=14), 1 M PBS (pH=7) and 0.5 M H_2_SO_4_ (pH=0) were used as electrolyte. The self-supporting electrode and carbon rod were used as the working electrode and counter electrode, respectively. Hg/HgO was used as the reference electrode when the electrolyte was 1 M KOH, and Ag/AgCl was used as the reference electrode when the electrolytes were 1 M PBS and 0.5 M H_2_SO_4_. All potentials were converted to Reversible Hydrogen Electrode (RHE) with the formula of E_RHE_ = E_Hg/HgO_ + 0.0592 × pH + 0.098 and E_RHE_= E_Ag/AgCl_ + 0.0591 × pH + 0.197. The linear sweep voltammetry (LSV) curves for HER were recorded at the potential range of -0.6~0.2 V vs. RHE with a scanning rate of 5 mV/s in 0.5 M H_2_SO_4_, 1 M PBS and 1 M KOH. All the LSV curves were corrected by 85% IR compensation. Tafel slopes were calculated based on the LSV curves. The mass activities of 40% Pt/C and Pt-CoP were normalized to Pt loading mass. The electrochemical impedance spectroscopy (EIS) tests were measured with frequencies ranging from 100 kHz to 0.1 with an AC amplitude of 5 mV at an overpotential 73 mV (vs. RHE, 0.5 M H_2_SO_4_), or 35 mV (vs. RHE, in 1 M KOH), or 89 mV (vs. RHE, in 1 M PBS). Chronopotentiometric curves were recorded for the long-term stability testing at 100 mA cm^-2^ (1 M KOH, 0.5 M H_2_SO_4_) and 10 mA cm^-2^ (1 M PBS). The electrochemical C_dl_ of the catalysts was determined from the double-layer charging curves derived from the cyclic voltammetry, different scan rates of CV including 20 mV/s, 40 mV/s, 60 mV/s, 80 mV/s, and 100 mV/s under the different voltage window in HER with full pH value. Then linear fitting of the charging current density differences Δj = (j_a_ - j_c_)/2 against the scan rate was done to fit the slope, and the slope is the double-layer capacitance C_dl_. Before testing, all electrocatalysts were activated by CV in the corresponding LSV range to exclude the influence of surface reconstruction or early phase transition during the HER process.

**1.8 Theoretical calculation method**

Our calculations utilizing spin-polarized density functional theory (DFT) were conducted using the Vienna ab initio simulation package (VASP), which employs a plane-wave basis set in conjunction with the projector augmented-wave method.^[1-3]^ The exchange-correlation potential in our analysis was addressed through the application of a generalized gradient approximation (GGA), specifically utilizing the Perdew-Burke-Ernzerhof (PBE) parametrization.^[4]^ Additionally, we incorporated the van der Waals correction as outlined in Grimme’s DFT-D3 model.^[5]^ A vacuum space of approximately 18 Å was implemented to mitigate any interactions between neighboring images. The energy cutoff was established at 450 eV. For the Brillouin-zone integration, a Γ-centered Monkhorst-Pack mesh with a density of 2 × 2 × 1 was employed. The structures underwent full relaxation, ensuring that the maximum force exerted on each atom was below 0.02 eV/Å, and the energy convergence criterion was set to a stringent 10^-5^ eV.^[6]^ Modeling is based on the CoP optimal crystal plane (111). The calculation models of Pt/C, CoP, and Pt-CoP are shown in Figure S1.

**2. Supporting Figure**

**
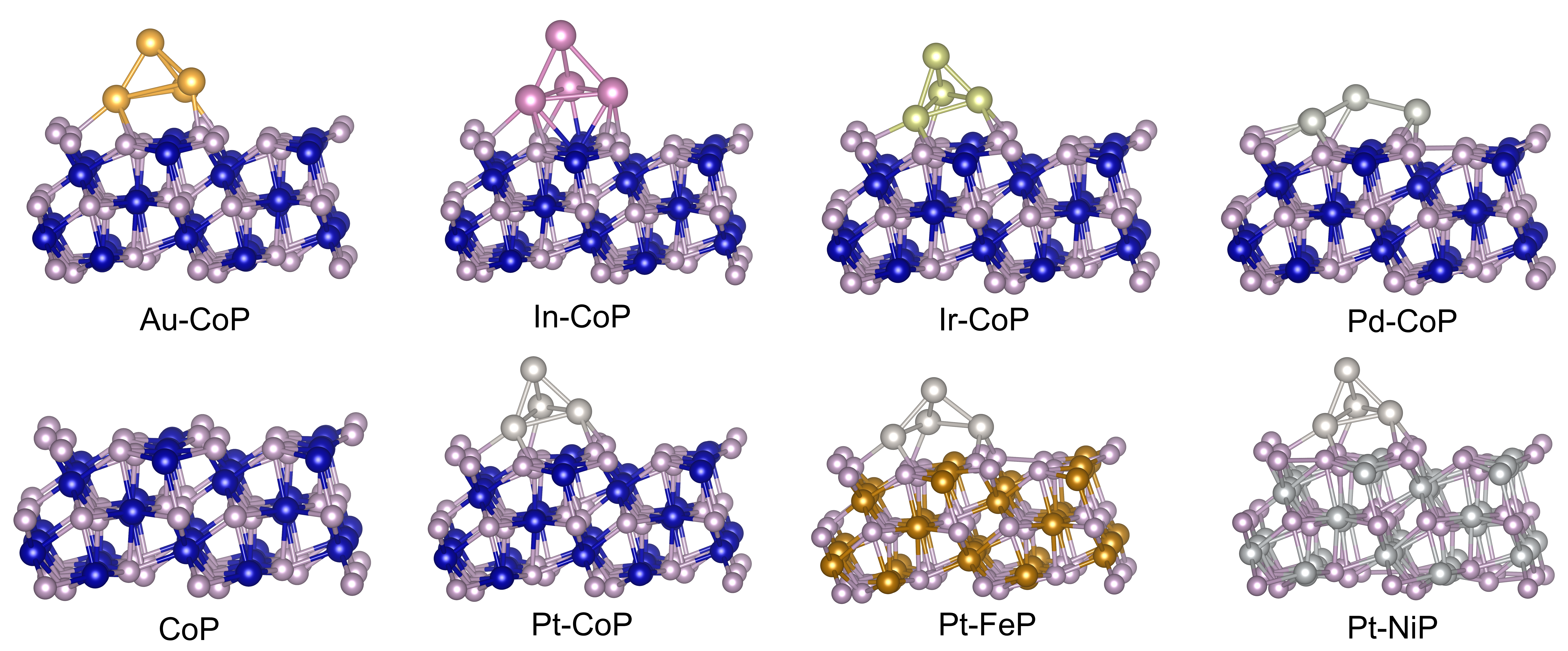
**

Figure S1. The geometric electronic structure of Au-CoP, In-CoP, Ir-CoP, Pd-CoP, CoP, Pt-CoP, Pt-FeP and Pt-NiP.


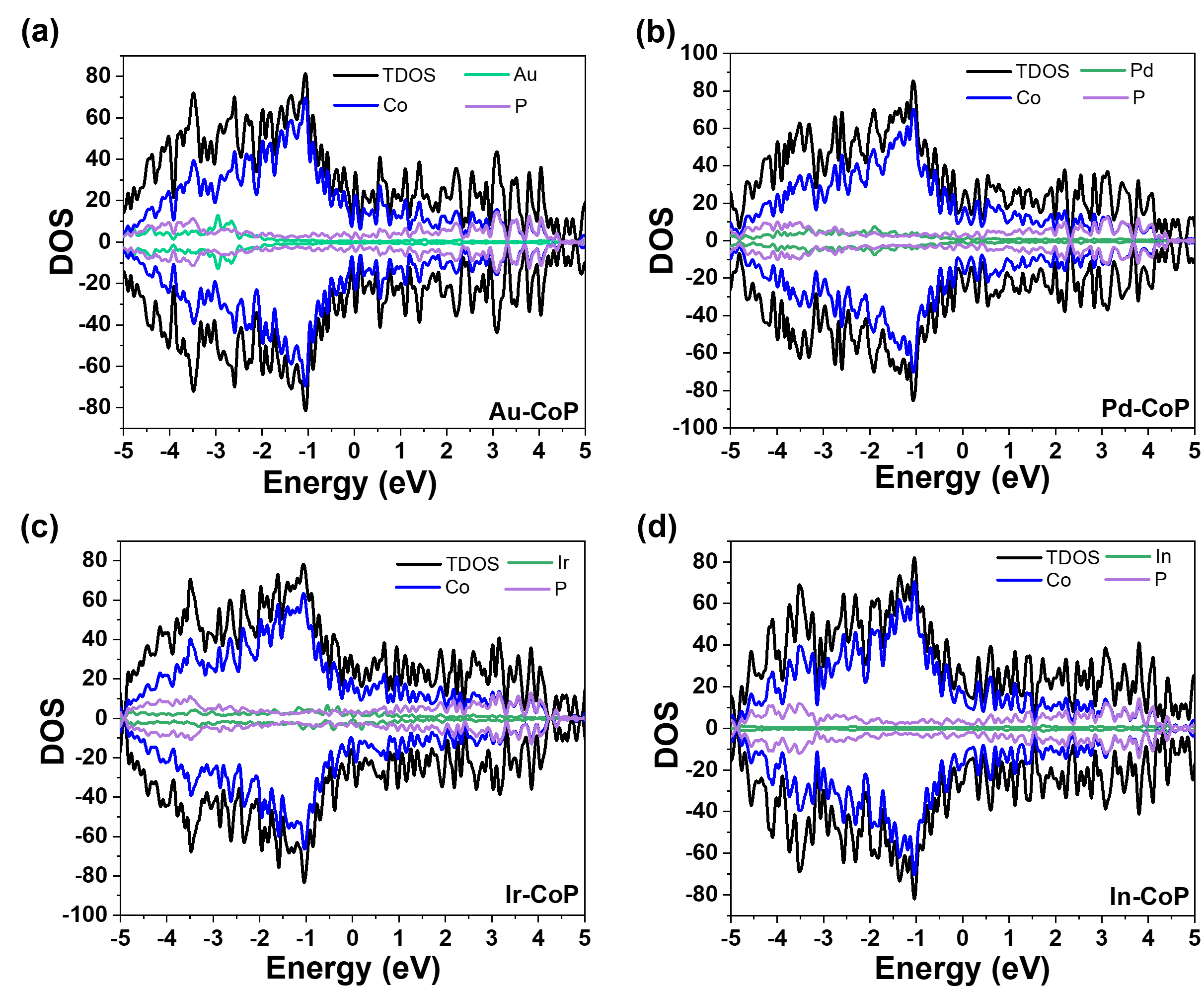


Figure S2. Densities of states (DOS) for Au-CoP, Pd-CoP, Ir-CoP, and In-CoP models.


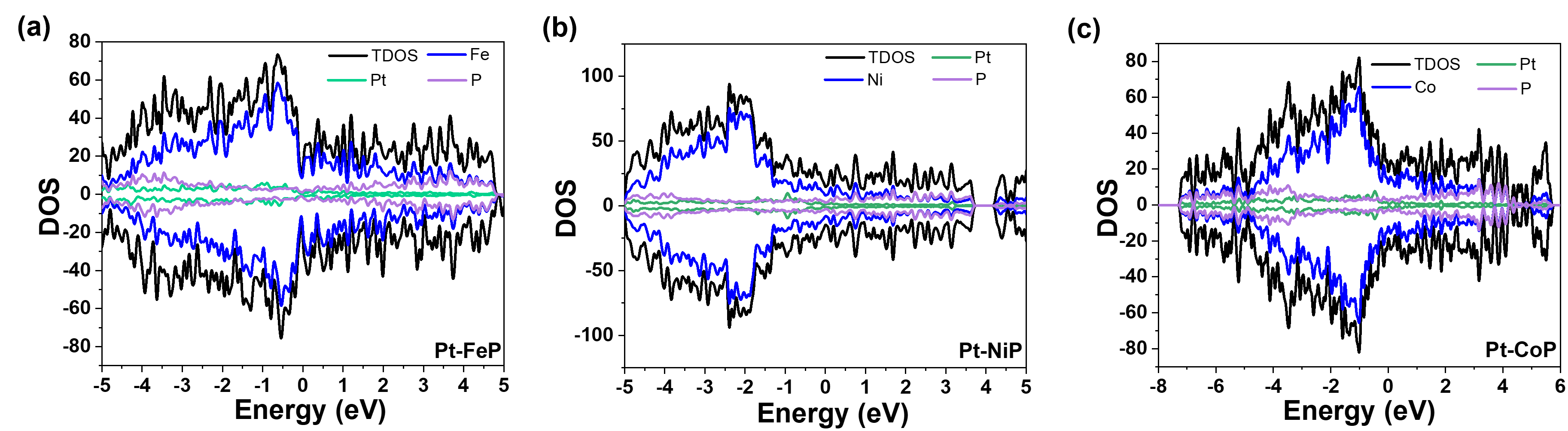


Figure S3. Densities of states (DOS) for Pt-FeP, Pt-NiP, and Pt-CoP models.

**
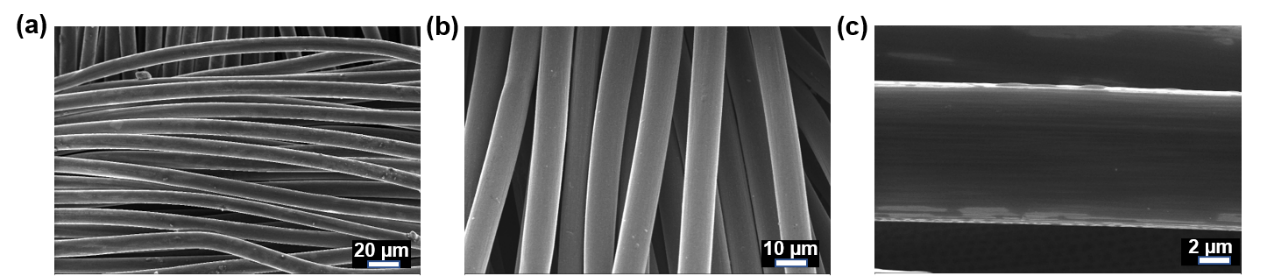
**

Figure S4. (a-c) The SEM images of CC at different resolutions.

**
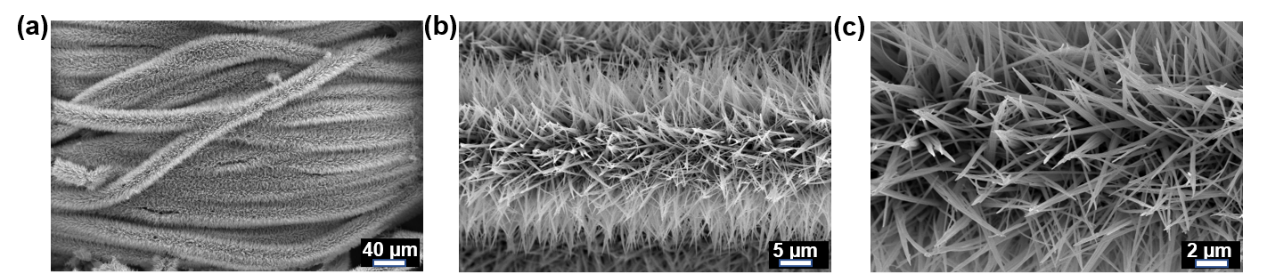
**

Figure S5. (a-c) The SEM images of Co(OH)F at different resolutions.


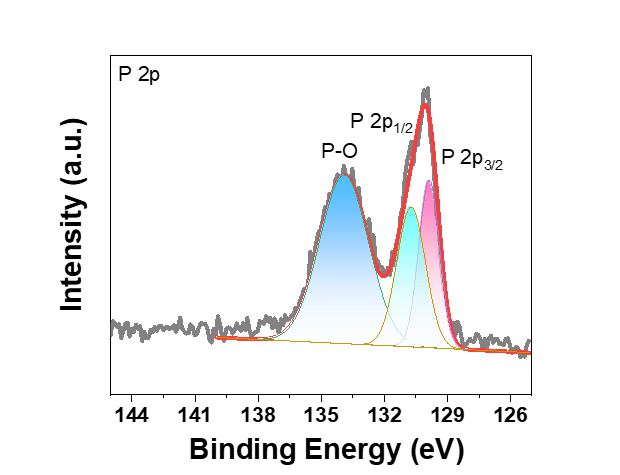


Figure S6. High-resolution XPS spectra of the P 2p region for CoP, subjected to CV in the absence of the Pt precursor solution.

**
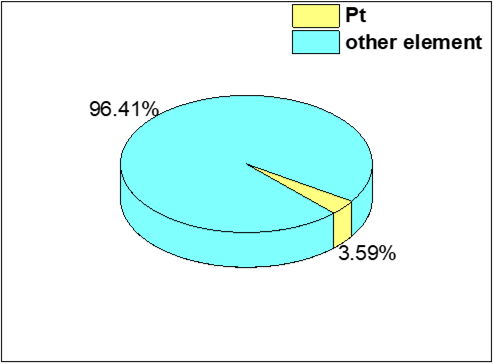
**

Figure S7. The inductively coupled plasma-mass spectrometry result.

**
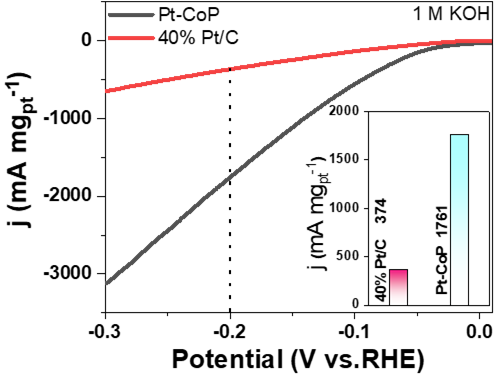
**

Figure S8. The HER LSVs in 1 M KOH solution and the mass activity of Pt-CoP and 40% Pt/C are normalized by the content of Pt, and the insert is current density comparison at -0.2 V.

**
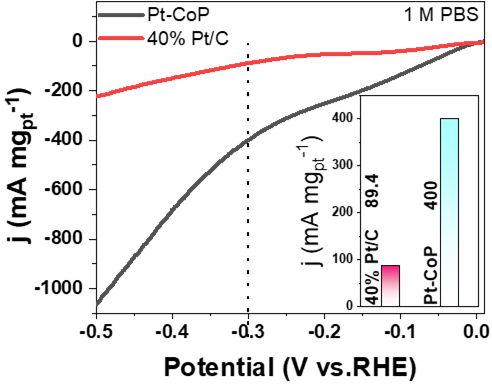
**

Figure S9. The HER LSVs in 1 M PBS solution and the mass activity of Pt-CoP and 40% Pt/C are normalized by the content of Pt, and the insert is current density comparison at -0.3 V.


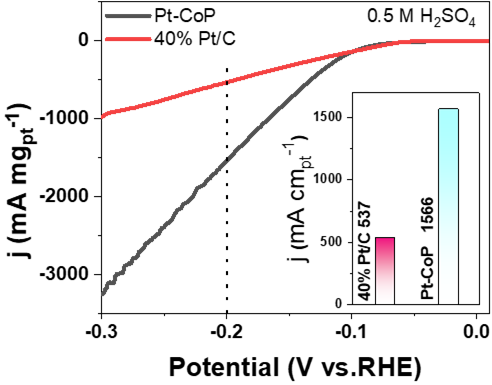


Figure S10. The HER LSVs in 0.5 H_2_SO_4_ solution and the mass activity of Pt-CoP and 40% Pt/C are normalized by the content of Pt, and the insert is current density comparison at -0.2 V.


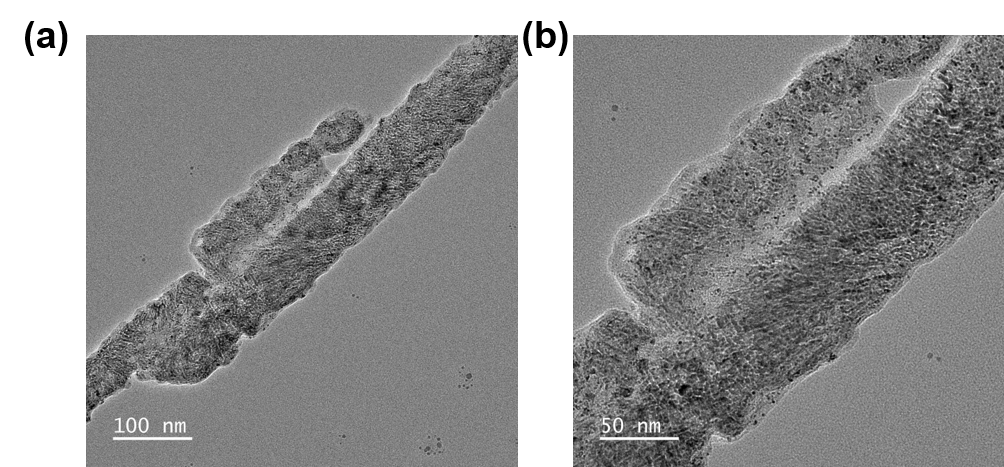


Figure S11. The TEM images of Pt-CoP sample after stability testing at a constant current density of 50 mA cm^-2^ for 50 hours.


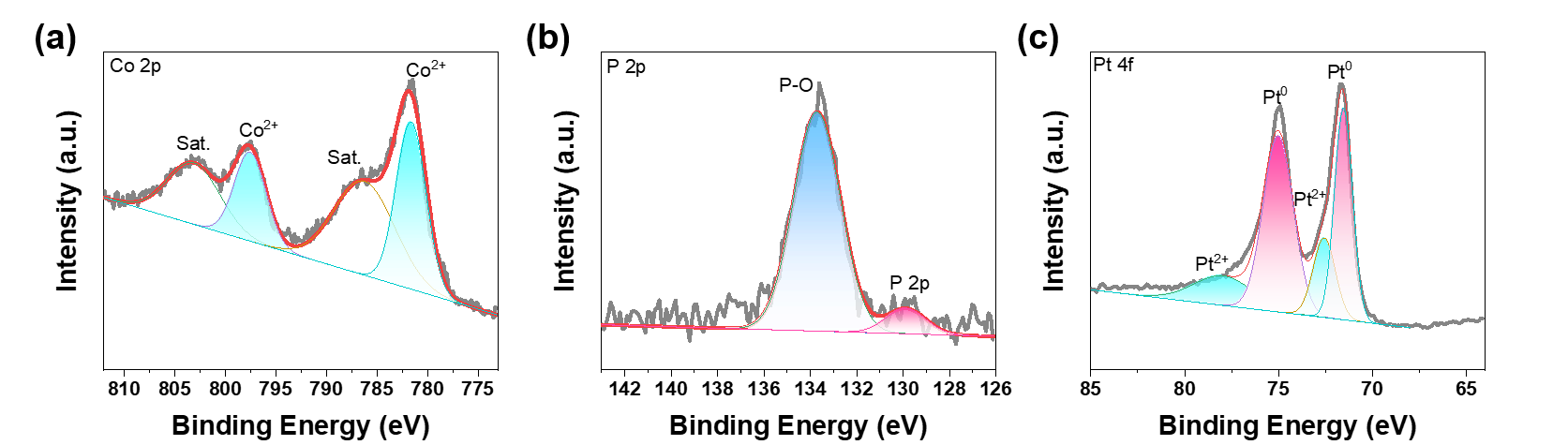


Figure S12. High-resolution XPS spectra of (a) Co 2p, (b) P 2p, and (c) Pt 4f for Pt-CoP sample after stability testing at a constant current density of 50 mA cm^-2^ for 50 hours.

**
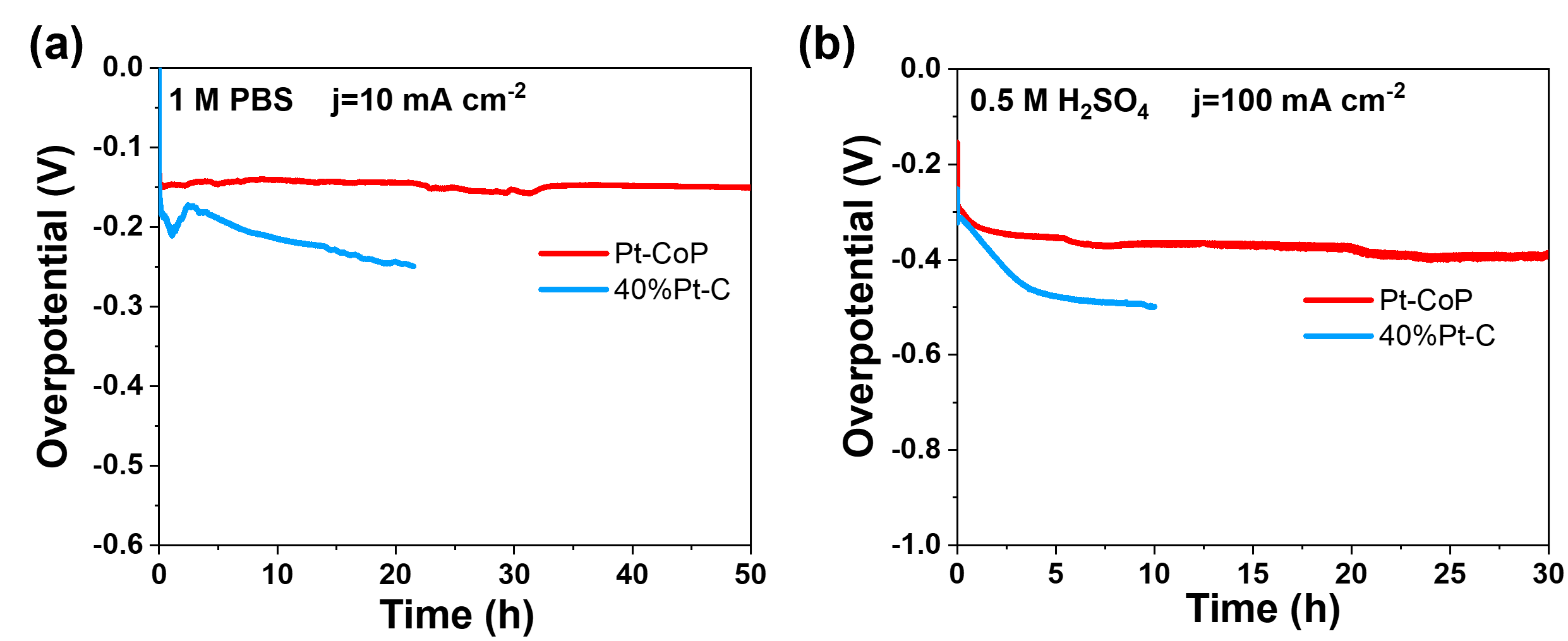
**

Figure S13. (a) Chronopotentiometric curves at 10 mA cm^-2^ in 1 M PBS. (b) Chronopotentiometric curves at 100 mA cm^-2^ in 0.5 M H_2_SO_4_ solution. The initial voltage fluctuations observed within the first few seconds of the test are primarily attributed to the transient instability of the testing system, which arises from the sudden application of the applied current.

**Table S1.** Comparison of HER activity of Pt-CoP with other reported Pt-based catalysts in alkaline electrolyte.

| **N** | **Catalyst** | **Electrolyte** | **HER overpotential at**  **10 mA cm^-2^** | **Ref.** |
| --- | --- | --- | --- | --- |
| 1 | Pt-CoP | 1 M KOH | 18 | This work |
| 2 | Pt_SA_/α-MoC_1−x_@C | 1 M KOH | 21 | ^[7]^ |
| 3 | PtCo@PtSn | 1 M KOH | 25 | ^[8]^ |
| 4 | PtCu-MoO_2_@C | 1 M KOH | 24 | ^[9]^ |
| 5 | Pt-MoS_2_/MWCNTs | 1 M KOH | 75 | ^[10]^ |
| 6 | PtCoMo@NC | 1 M KOH | 51 | ^[11]^ |
| 7 | Pt-TiO_2−x_NSs | 1 M KOH | 69 | ^[12]^ |
| 8 | GaPt_3_ | 1 M KOH | 48 | ^[13]^ |
| 9 | Pt_5_/HMCS | 1 M KOH | 46.2 | ^[14]^ |
| 10 | CDs/Pt-PANI | 1 M KOH | 56 | ^[15]^ |
| 11 | Pt@PCM | 1 M KOH | 150 | ^[16]^ |
| 12 | Pt_3_Co@NCNT | 1 M KOH | 36 | ^[17]^ |

**Table S2.** Comparison of HER activity of Pt-CoP with other reported Pt-based catalysts in neutral electrolyte.

| **N** | **Catalyst** | **Electrolyte** | **HER overpotential at**  **10 mA cm^-2^** | **Ref.** |
| --- | --- | --- | --- | --- |
| 1 | Pt-CoP | 1 M PBS | 26.3 | This work |
| 2 | Pt_SA_/α-MoC_1−x_@C | 1 M PBS | 36 | ^[7]^ |
| 3 | PtCu-MoO_2_@C | 1 M PBS | 18 | ^[8]^ |
| 4 | RuCoP | 1 M PBS | 131 | ^[9]^ |
| 5 | Pt-MoS_2_/MWCNTs | 1 M PBS | 92 | ^[10]^ |
| 6 | PtCoMo@NC | 1 M PBS | 66 | ^[11]^ |
| 7 | Pt-TiO_2−x_NSs | 1 M PBS | 87 | ^[12]^ |
| 8 | GaPt_3_ | 1 M PBS | 103 | ^[13]^ |
| 9 | Mo_2_C@NC@Pt | 1 M PBS | 25 | ^[18]^ |
| 10 | PtRu/CC_1500_ | 1 M PBS | 25 | ^[19]^ |
| 11 | Pt-IrO_2_/CC | 1 M PBS | 26 | ^[20]^ |
| 12 | Pt/np-Co_0.85_Se | 1 M PBS | 55 | ^[21]^ |

**Table S3.** Comparison of HER activity of Pt-CoP with other reported Pt-based catalysts in acidic electrolyte.

| **N** | **Catalyst** | **Electrolyte** | **HER overpotential at**  **10 mA cm^-2^** | **Ref.** |
| --- | --- | --- | --- | --- |
| 1 | Pt-CoP | 0.5 M H_2_SO_4_ | 79 | This work |
| 2 | Pt_6_Mn | 0.5 M H_2_SO_4_ | 39 | ^[22]^ |
| 3 | Ni–Pt films | 0.5 M H_2_SO_4_ | 90 | ^[23]^ |
| 4 | PtCu-MoO_2_@C | 0.5 M H_2_SO_4_ | 42 | ^[9]^ |
| 5 | Pt-CNTs | 0.5 M H_2_SO_4_ | 41 | ^[24]^ |
| 6 | Pt_1_@Fe-N-C | 0.5 M H_2_SO_4_ | 60 | ^[25]^ |
| 7 | Pt-TiO_2−x_NSs | 0.5 M H_2_SO_4_ | 36 | ^[12]^ |
| 8 | Pt/VS_2_/CP | 0.5 M H_2_SO_4_ | 77 | ^[26]^ |
| 9 | Pt/LSG | 0.5 M H_2_SO_4_ | 131 | ^[27]^ |
| 10 | PtCoFe@Cn | 0.5 M H_2_SO_4_ | 45 | ^[28]^ |
| 11 | 50Pt/Mo_2_C | 0.5 M H_2_SO_4_ | 100 | ^[29]^ |
| 12 | Pt-SnS_2_ | 0.5 M H_2_SO_4_ | 117 | ^[30]^ |

**Reference**

[1] P. Hohenberg, W. Kohn, *Phys. Rev.* **1964**, 136, B864.

[2] W. Kohn, L. J. Sham, *Phys. Rev.* **965**, 140, A1133.

[3] G. Kresse, J. Furthmüller, *Phys. Rev. B* **1996**, 54, 11169.

[4] J. P. Perdew, K. Burke, M. Ernzerhof, *Phys. Rev. Lett.* **1996**, 77, 3865.

[5] S. Grimme, J. Antony, S. Ehrlich, H. Krieg, *J. Chem. Phys.* **2010**, 132.

[6] H. J. Monkhorst, J. D. Pack, *Phys. Rev. B* **1976**, 13, 5188.

[7] W. Wang, Y. Wu, Y. Lin, J. Yao, X. Wu, C. Wu, X. Zuo, Q. Yang, B. Ge, L. Yang, *Adv. Funct. Mater.* **2022**, 32, 2108464.

[8] J. Chen, G. Qian, H. Zhang, S. Feng, Y. Mo, L. Luo, S. Yin, *Adv. Funct. Mater.* **2022**, 32, 2107597.

[9] C. Zhang, P. Wang, W. Li, Z. Zhang, J. Zhu, Z. Pu, Y. Zhao, S. Mu, *J. Mater. Chem. A* **2020**, 8, 19348.

[10] A. Fan, P. Zheng, C. Qin, X. Zhang, X. Dai, D. Ren, X. Fang, C. Luan, J. Yang, *Electrochim. Acta* **2020**, 358, 136927.

[11] W.-H. Huang, X.-M. Li, D.-Y. Yu, X.-F. Yang, L.-F. Wang, P.-B. Liu, J. Zhang, *Nanoscale* **2020**, 12, 19804.

[12] K. M. Naik, E. Higuchi, H. Inoue, *Nanoscale* **2020**, 12, 11055.

[13] S.-C. Lim, C.-Y. Chan, K.-T. Chen, H.-Y. Tuan, *Electrochim. Acta* **2019**, 297, 288.

[14] X. K. Wan, H. B. Wu, B. Y. Guan, D. Luan, X. W. Lou, *Adv. Mater.* **2020**, 32, 1901349.

[15] Q. Dang, Y. Sun, X. Wang, W. Zhu, Y. Chen, F. Liao, H. Huang, M. Shao, *Appl. Catal., B* **2019**, 257, 117905.

[16] H. Zhang, P. An, W. Zhou, B. Y. Guan, P. Zhang, J. Dong, X. W. Lou, *Sci. Adv.* **2018**, 4, eaao6657.

[17] V. A. Saveleva, K. Ebner, L. Ni, G. Smolentsev, D. Klose, A. Zitolo, E. Marelli, J. Li, M. Medarde, O. V. Safonova, *Angew. Chem., Int. Ed.* **2021**, 60, 11707.

[18] J.-Q. Chi, J.-Y. Xie, W.-W. Zhang, B. Dong, J.-F. Qin, X.-Y. Zhang, J.-H. Lin, Y.-M. Chai, C.-G. Liu, *ACS Appl. Mater. Interfaces* **2019**, 11, 4047.

[19] L. Li, G. Zhang, B. Wang, T. Yang, S. Yang, *J. Mater. Chem. A* **2020**, 8, 2090.

[20] L. Li, B. Wang, G. Zhang, G. Yang, T. Yang, S. Yang, S. Yang, *Adv. Energy Mater.* **2020**, 10, 2001600.

[21] K. Jiang, B. Liu, M. Luo, S. Ning, M. Peng, Y. Zhao, Y.-R. Lu, T.-S. Chan, F. M. de Groot, Y. Tan, *Nat. Commun.* **2019**, 10, 1743.

[22] J. Hu, C. Fang, X. Jiang, D. Zhang, Z. Cui, *Inorg. Chem. Front.* **2020**, 7, 4377.

[23] K. Eiler, S. Suriñach, J. Sort, E. Pellicer, *Appl. Catal., B* **2020**, 265, 118597.

[24] W. Zhong, W. Tu, Z. Wang, Z. Lin, A. Xu, X. Ye, D. Chen, B. Xiao, *J. Energy Chem.* **2020**, 51, 280.

[25] X. Zeng, J. Shui, X. Liu, Q. Liu, Y. Li, J. Shang, L. Zheng, R. Yu, *Adv. Energy Mater.* **2018**, 8, 1701345.

[26] J. Zhu, L. Cai, X. Yin, Z. Wang, L. Zhang, H. Ma, Y. Ke, Y. Du, S. Xi, A. T. Wee, *ACS Nano* **2020**, 14, 5600.

[27] P. Nayak, Q. Jiang, N. Kurra, X. Wang, U. Buttner, H. N. Alshareef, *J. Mater. Chem. A* **2017**, 5, 20422.

[28] J. Chen, Y. Yang, J. Su, P. Jiang, G. Xia, Q. Chen, *ACS Appl. Mater. Interfaces* **2017**, 9, 3596.

[29] S. Saha, B. Martin, B. Leonard, D. Li, *J. Mater. Chem. A* **2016**, 4, 9253.

[30] G. Liu, Y. Qiu, Z. Wang, J. Zhang, X. Chen, M. Dai, D. Jia, Y. Zhou, Z. Li, P. Hu, *ACS Appl. Mater. Interfaces* **2017**, 9, 37750.
